# Robotic Hybrid Ablation for Persistent Atrial Fibrillation With Diffuse Pericardial Adhesions

**DOI:** 10.1016/j.jaccas.2026.110211

**Published:** 2026-09-04

**Authors:** Wong Max Kwun Hung, Inderjeet Bhatia, Wan Anakin Chi Hong, Fan Katherine Yue Yan, Ling Ian Wood Hay, Daniel Tai Leung Chan

**Affiliations:** a Department of Cardiothoracic Surgery, Queen Mary Hospital, Hong Kong SAR, China; b School of Clinical Medicine, The University of Hong Kong, Hong Kong SAR, China; c Cardiac Medical Unit, Grantham Hospital, Grantham, Hong Kong SAR, China; d Li Ka Shing Faculty of Medicine, Department of Surgery, School of Clinical Medicine, The University of Hong Kong, Queen Mary Hospital, Hong Kong SAR, China

**Keywords:** ablation, atrial fibrillation, robotic surgery

## Abstract

**Background:**

Atrial fibrillation (AF), the most prevalent arrhythmia, poses significant health risks globally, particularly in aging populations. For symptomatic persistent AF resistant to standard treatments, the guidelines recommend hybrid approaches. Convergent hybrid ablation with robotic epicardial surgery enhances access in cases with challenging pericardial adhesions.

**Case Summary:**

We present a case of persistent AF with past catheter ablation complicated by pericardial effusion now presenting with worsening symptoms. Given anticipated pericardial adhesions, convergent hybrid ablation with a robotic-assisted epicardial component was performed, followed by a successful second-stage endocardial ablation. The patient had an uneventful recovery and remains in sinus rhythm.

**Discussion:**

This report demonstrates the successful application of a robotic platform to the epicardial component of a Convergent hybrid ablation procedure, in which diffuse adhesions precluded the standard approach.

**Take-Home Message:**

Robotic-assisted hybrid ablation is a novel approach for hybrid ablation for long-standing persistent AF, especially in patients with pericardial adhesions.

## Visual Summary

**Figure:**
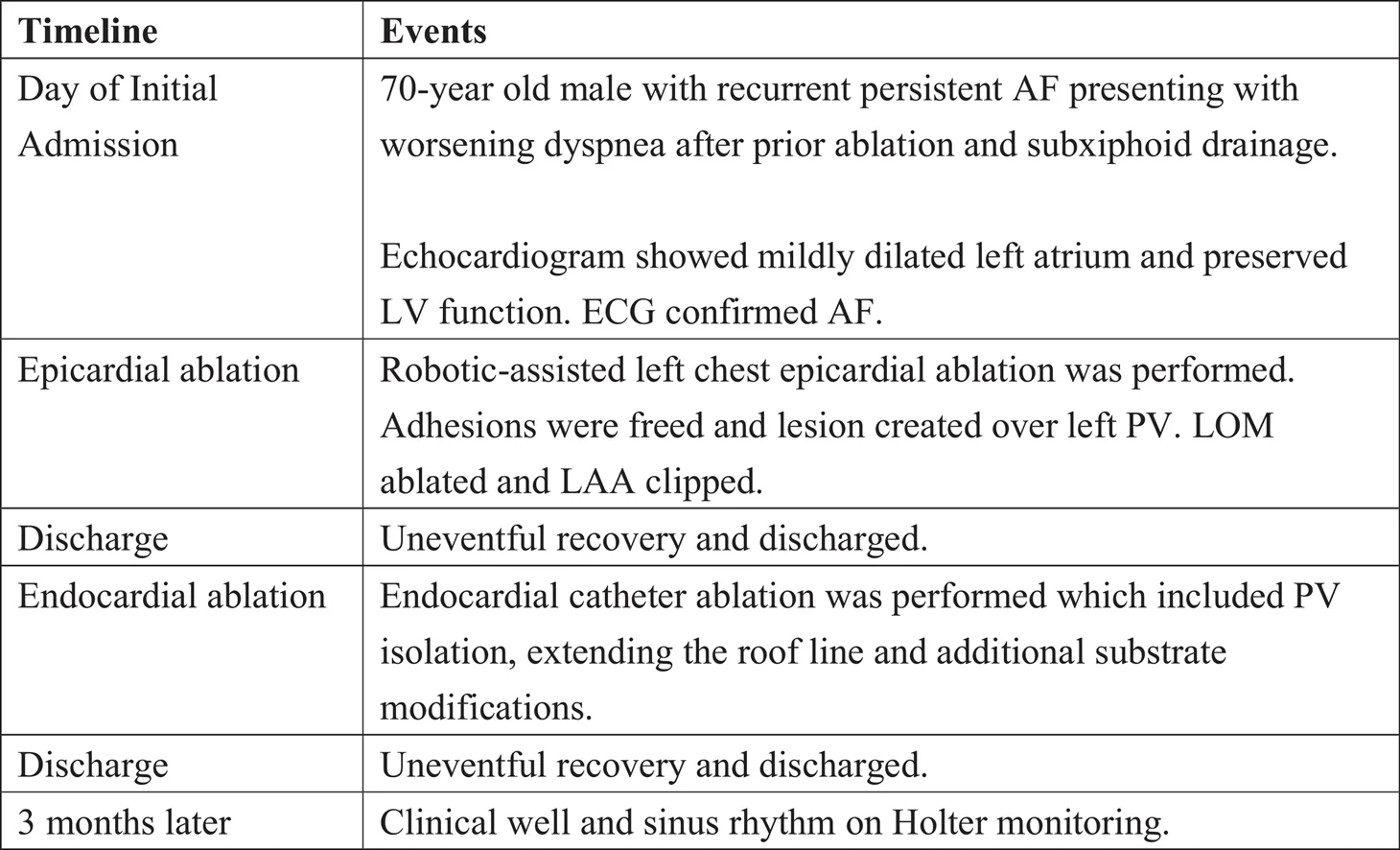

## History of Presentation and Past Medical History

A 70-year-old man with long-standing atrial fibrillation (AF) had undergone catheter ablation in 1998, which was complicated by pericardial effusion requiring surgical subxiphoid drainage. He underwent another catheter ablation the following year for supraventricular arrhythmia; thereafter, he subsequently returned to sinus rhythm. In 2019, he had a breakthrough AF which required electrical cardioversion. A routine electrocardiogram in 2022 confirmed sinus rhythm, but in 2024, he began developing chest pain and dyspnea, and persistent AF had returned. Over the next 6 months, his dyspnea worsened; he was in NYHA functional class II-III and was started on anticoagulation because of a high CHA_2_DS_2_-VASc score of 4. His other medical history included hypertension, hyperlipidemia, obstructive sleep apnea, and ischemic heart disease with coronary stenting performed in 2019.

## Differential Diagnosis

The patient's symptoms initially raised suspicion for underlying ischemic heart disease or cardiomyopathy. However, these differentials were successfully excluded following a normal coronary computed tomography angiography and an echocardiogram.

## Investigations

Echocardiography showed satisfactory left ventricular contraction with a mildly dilated left atrium, and an electrocardiogram confirmed he was in AF.

He was subsequently referred to our center to be considered for hybrid ablation. However, it was anticipated that adhesions from the previous subxiphoid drainage would make the standard subxiphoid approach difficult. Therefore, the epicardial component utilized robotic assistance, with port access from the left chest only, rather than the xiphoid incision.

## Management

The robotic epicardial component was performed in July 2025 with the Da Vinci Xi system (Intuitive Inc). The patient was put under general anesthesia with single-lumen intubation and a bronchial blocker to isolate the left lung. Transesophageal echocardiogram revealed no clots in the left atrial appendage (LAA). The patient's left chest was slightly elevated, and 4 ports were inserted: a camera port at the fourth intercostal space (ICS) along the midaxillary line; left and right robotic arm ports at the third and sixth ICS, respectively, along the anterior axillary line; and a 12 mm working port at the sixth ICS along the midaxillary line (Figure 1). The thoracic cavity was then insufflated with CO_2_ and the robotic system was docked.

**Figure 1:**
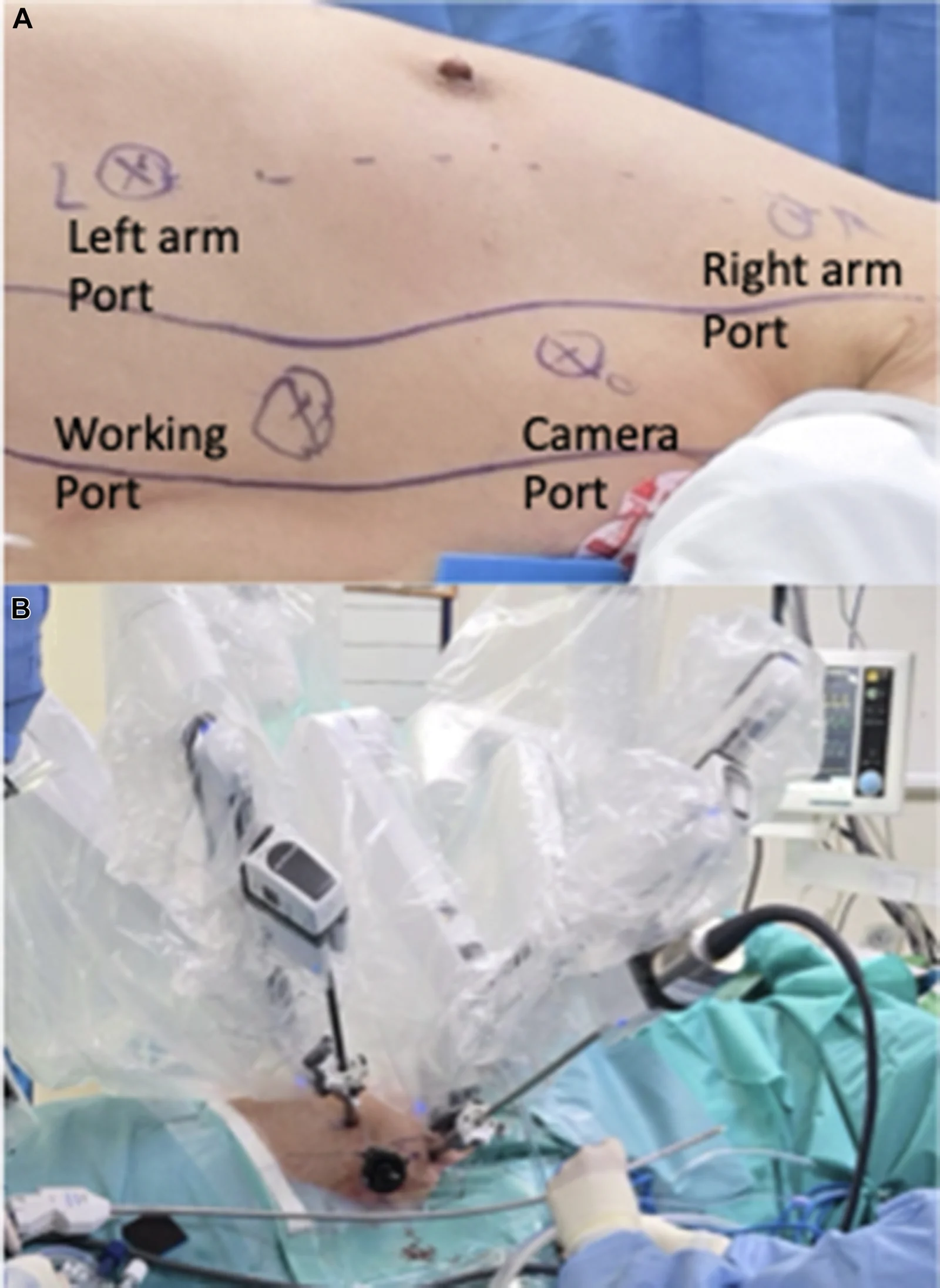
Setup of Da Vinci Xi System (Intuitive Inc) (A) Marking of patient's left chest – camera port at fourth intercostal space (ICS), above midaxillary line, 12 mm working port at sixth ICS, same level. Right arm port at second ICS, above anterior axillary line, and left arm at sixth ICS, same level. (B) After robotic arms dock in.

The left phrenic nerve was visualized, and the pericardium was opened inferior to it, revealing diffuse mild-to-moderate pericardial adhesions which were subsequently freed (Figure 2). Epicardial mapping was performed to generate a 3-dimensional electroanatomic map (Figure 3). After mapping, the ligament of Marshall (LOM) was visualized and cut. The EPi-Sense coagulation device (AtriCure) was inserted via the 12 mm port, and ablation lesions were created over the LOM, along the anterior lines of the left pulmonary veins (PVs) and on the posterior wall between the right and left inferior PVs. Each lesion was double-tapped (ie, a total of 180 seconds) at an energy setting of 60 W. Esophageal temperature was monitored to avoid exceeding 38 ^o^C or a 1 ^o^C rapid rise. Repeat mapping after ablation showed no electrical activity in the targeted areas (Figures 4A and 4B). An AtriClip Pro 2 45 mm device (AtriCure) was then applied to the LAA. Transesophageal echocardiogram confirmed no residual flow and a 4 mm stump (Figure 5). Direct-current cardioversion restored sinus rhythm at the end of the procedure.

**Figure 2:**
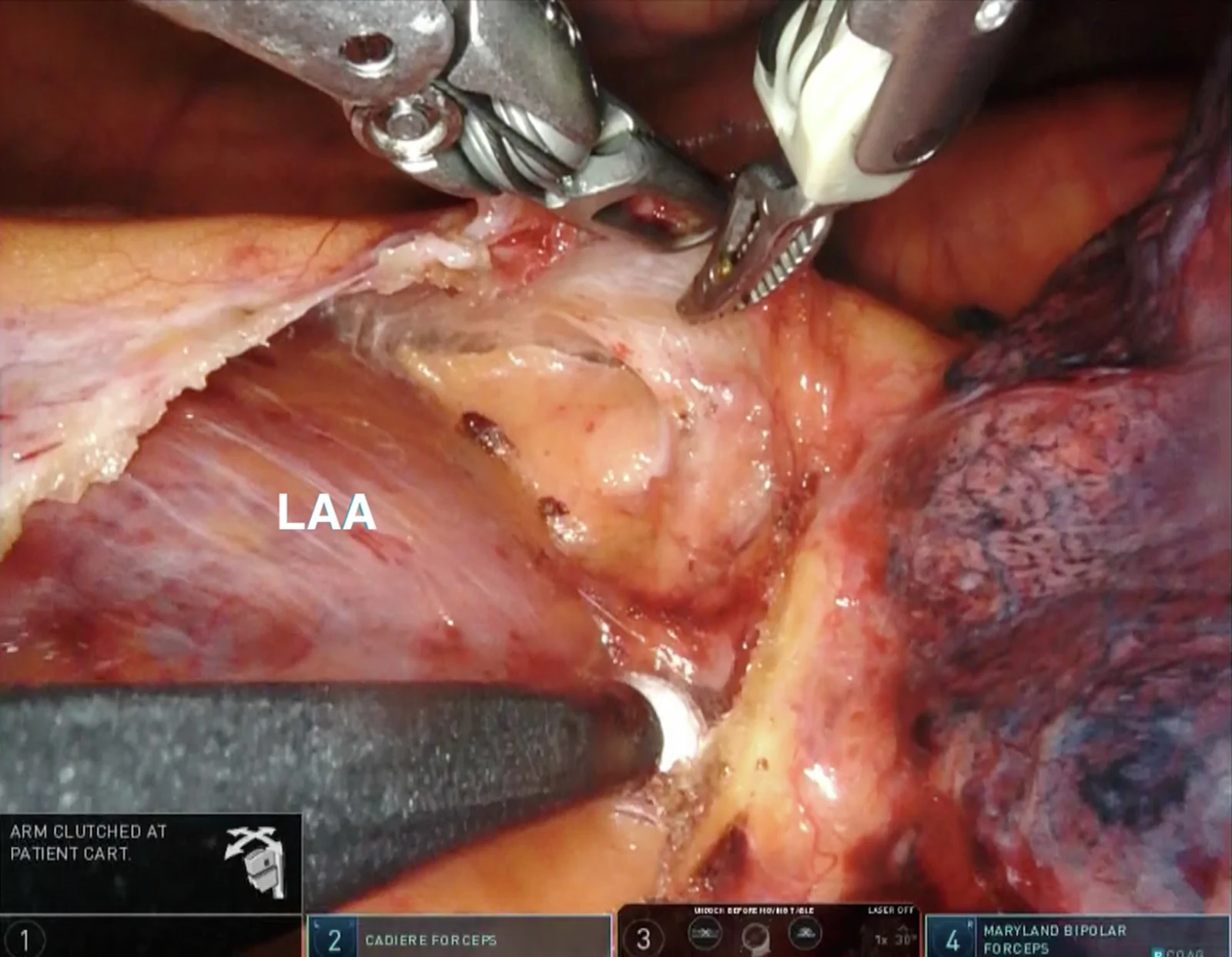
Pericardial Adhesions Diffuse adhesions inside the pericardial space after opening the pericardium. LAA = left atrial appendage.

**Figure 3:**
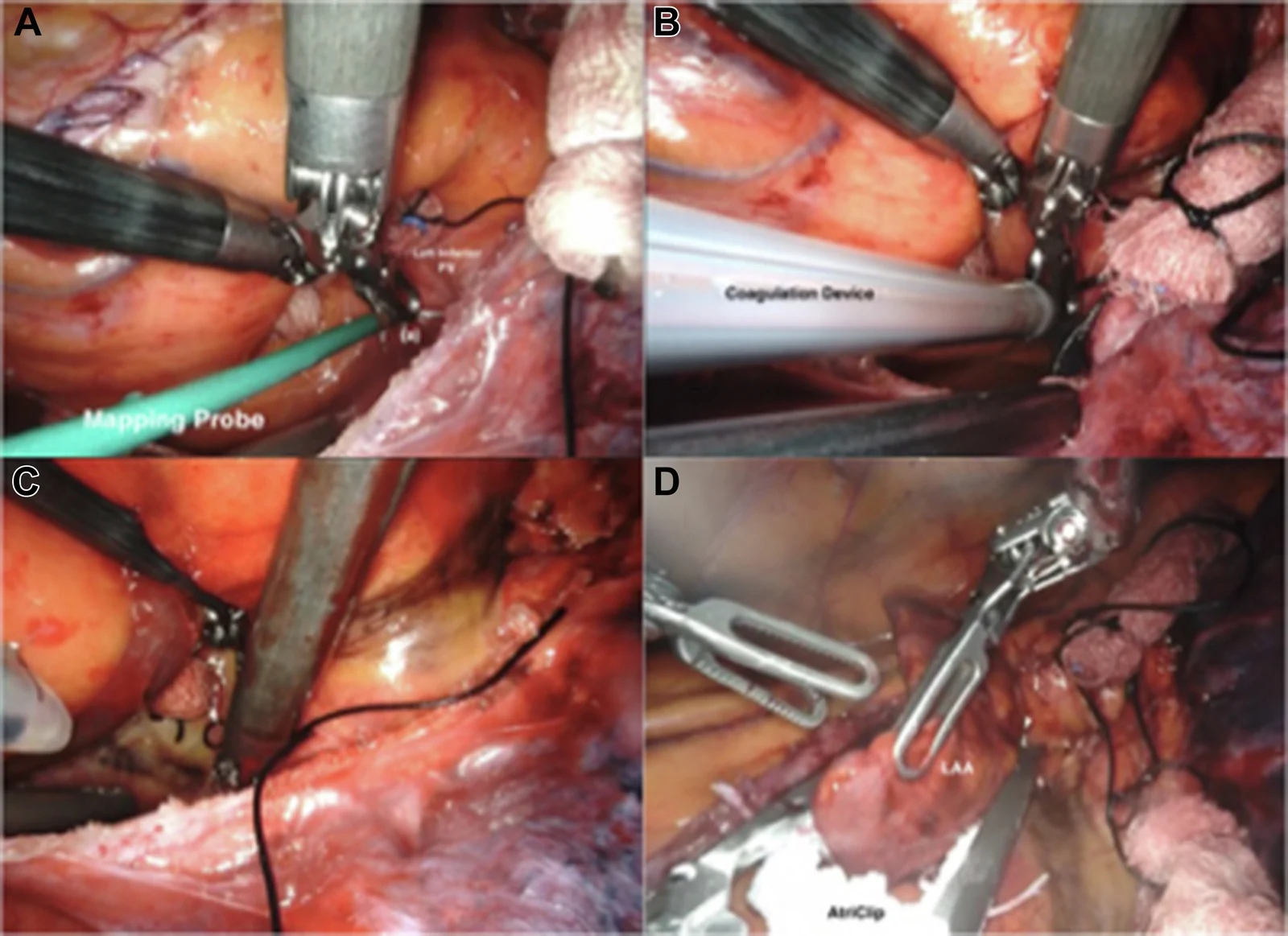
The Ablation Procedure (A) Epicardial mapping probe placed inside the oblique sinus. (B) Epi-sense Coagulation device in the oblique sinus ablating the posterior wall. (C) The char-looking posterior wall after ablation. (D) Left atrial appendage clip in place before closure.

**Figure 4:**
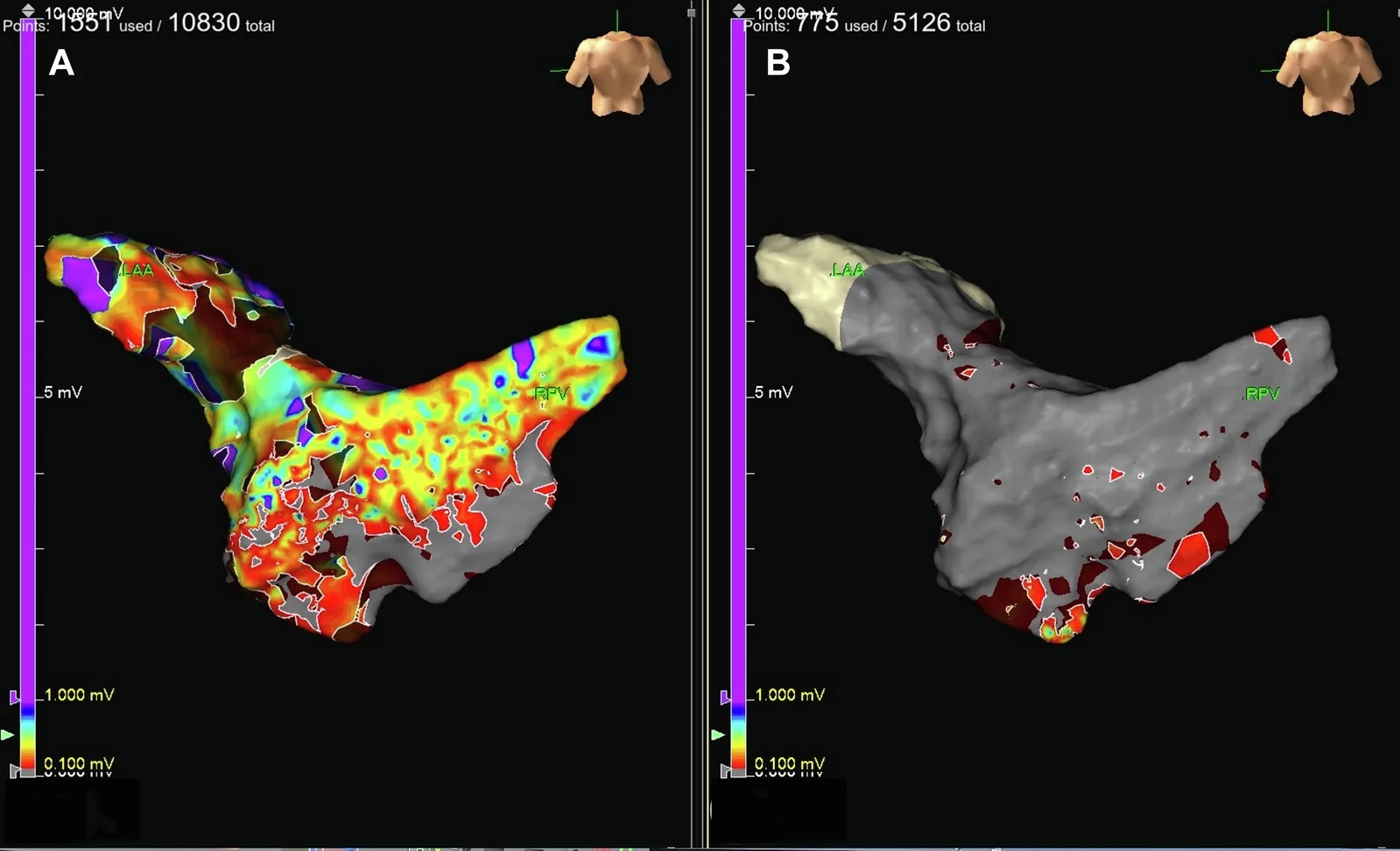
Electrical Mapping of Left Atrial Posterior Wall Before and After Epicardial Ablation (A) Electrically active regions at baseline. (B) Electrically silent (gray) regions following the procedure.

**Figure 5:**
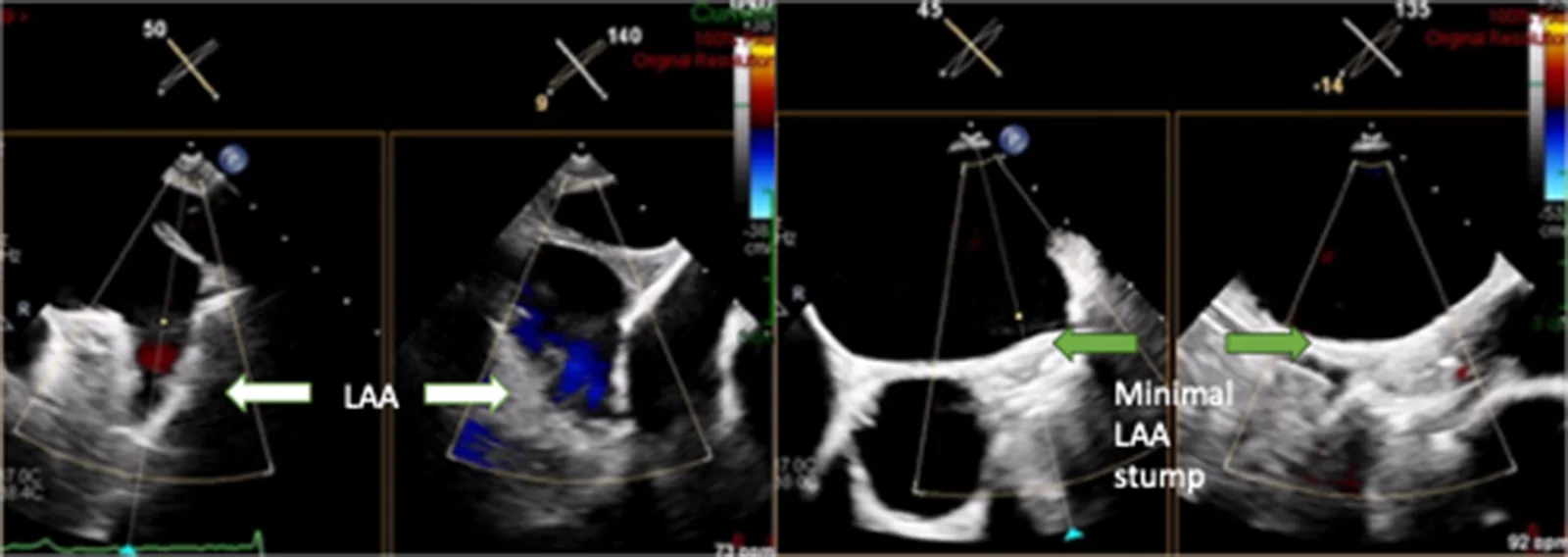
Preoperative and Postoperative LAA TEE Transesophageal echocardiogram before operation (left) showing no left atrial appendage (LAA) clot. Transesophageal echocardiogram after operation (right) showing closed LAA with no residual blood flow and minimal (∼4 mm) LAA stump.

The total operative time was 186 minutes. The patient was extubated on the table and was observed in the intensive care unit overnight. He recovered well without any major complications. No blood transfusion was required. The chest drain was removed on postoperative day 2, and he was discharged home on postoperative day 3.

After 8 weeks, the patient remained in sinus rhythm and was brought to the Cardiac Catheterization laboratory for the endocardial component of the Convergent procedure. After standard vascular access and transseptal puncture to the left atrium, mapping was performed using the CARTO system (Biosense Webster Inc) (Figure 6A). This confirmed postepicardial ablation scarring on the left atrial floor, connecting to the inferior PVs and forming a complete floor line. Subsequently, PV isolation was performed using wide antral circumferential ablation, achieving isolation. Finally, the roof line was extended anteriorly by ablation at the base of the LAA and at sites with complex fractionated atrial electrograms over the anteroseptum (Figure 6B).

**Figure 6:**
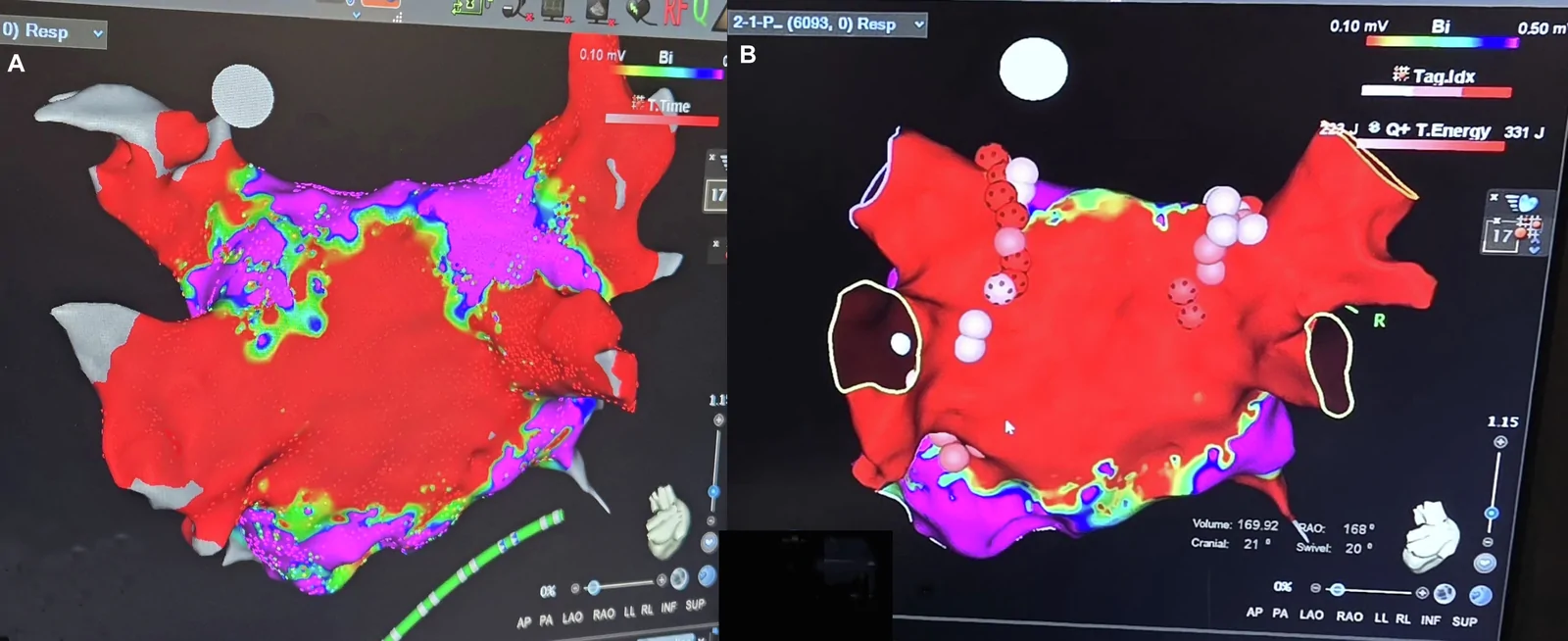
Electrical Mapping of Left Atrial Posterior Wall Before and After Endocardial Ablation (A) Electrically active regions at baseline, with previous epicardial ablation regions remaining silent. (B) Electrical silence (red) achieved at the pulmonary vein and posterior wall following the procedure. AP = accessory pathway; PA = pulmonary artery; INF = inferior; LAO = left anterior oblique; LL = left lateral; RAO = right anterior oblique; RL = right lateral.

The endocardial ablation was successfully performed in 240 minutes without major complications.

The patient had an uneventful recovery and was discharged on postoperative day 2 with his usual anticoagulants and diuretic agents.

## Outcome and Follow-Up

At the 3-month follow-up, a 24-hour Holter showed that he remained in sinus rhythm with no sustained arrhythmias. A 3.8-second pause occurred due to a blocked premature atrial contraction. Otherwise, he is recovering well and continues to take his usual medications, including a beta-blocker, an anticoagulant, and a diuretic.

## Discussion

This case demonstrates the successful use of a robotic-assisted approach for the epicardial component of hybrid ablation in a patient with persistent AF. To our knowledge, this case is the first report of such an approach in Asia.

Historically, the Cox-Maze procedures, developed since 1987, formed the cornerstone of surgical therapy for AF because of their high efficacy.^1^ However, the traditional Cox-Maze procedure is invasive and requires cardiopulmonary bypass. Therefore, the popularity of minimally invasive strategies, such as catheter-based or video-assisted thoracoscopic surgery approaches, continues to rise. However, the efficacy of catheter-based strategies is limited by incomplete substrate modification and an inability to ablate certain anatomical targets. Although video-assisted thoracoscopic surgery ablation provides improved access, it is associated with a high complication rate and may not reliably create transmural lesions.^2^ Therefore, hybrid convergent procedures have become popular because they combine the merits of both techniques and are complementary. This multidisciplinary approach, involving surgeons, electrophysiologists, and cardiologists, has proved effective. Independent studies from centers across Europe^3^^,^^4^ and the United States^5^, ^6^, ^7^ have demonstrated the efficacy of hybrid convergent procedures for the treatment of persistent AF. This culminated in the multicenter CONVERGE (Convergence of Epicardial and Endocardial Ablation for the Treatment of Symptomatic Persistent AF) trial, which showed that a hybrid approach is superior to catheter ablation alone.^8^ Nevertheless, particularly in persistent AF, PV isolation alone may not be sufficient to achieve full freedom from AF, as additional AF substrates may include the LAA, the left atrial posterior wall, and the LOM. Khalpey et al. first described the “Robotic Convergent Plus” procedure, which uses a robotic platform and extends the lesion set to include the left atrial posterior wall, excludes the LAA, and dissects the LOM.^9^ They, and subsequently other centers, found that, compared with a nonrobotic subxiphoid approach, patients experience better postoperative recovery and good efficacy for the treatment of persistent AF.^10^

Compared to the xiphoid VAT approach, the robotic-assisted approach offers several advantages, including superior visualization, improved capability to address difficult anatomy, such as pericardial adhesions, and better access in high-spine regions, enabling more comprehensive lesion creation. The versatile robotic arms provide stability and dexterity that could greatly facilitate the dissection of pericardial adhesions, especially at the posterior wall in the oblique sinus, where endoscopic visualization can be challenging. A left-chest approach enables the creation of ablation lines anterior to the left PV and around the LOM. This may further simplify and shorten the second-stage catheter ablation. The robotic platform addresses the technical difficulty of treating “high spine” anatomy (reported to be common in Asia). This is because the robotic approach elevates the LA wall and visualizes it from the left side, whereas an ablation catheter via a xiphoid approach may not be able to reach the high spine area, usually near the left PV.

## Conclusions

In hybrid management of persistent/long-standing AF, a unilateral robotic-assisted epicardial ablation was a feasible alternative to the standard subxiphoid pericardioscopic approach.

Robotic surgery offers several advantages: superior dexterity, superior anatomical exposure, which enables the creation of more ablation lesions, and improved visualization of the high spine area. Thus, this has proven that robotic-assisted epicardial ablation is safe even in the presence of diffuse pericardial adhesions.

## Funding Support and Author Disclosures

Dr Chan has served as a clinical proctor for AtriCure Inc for the past year. All other authors have reported that they have no relationships relevant to the contents of this paper to disclose.

**Take-Home Message**

• Robotic-assisted hybrid ablation is a novel approach for hybrid ablation for long-standing persistent AF, especially in patients with pericardial adhesions.

**Visual Summary:** Timeline of the Case

Timeline	Events
Day of initial admission	70-y old man with recurrent persistent AF presenting with worsening dyspnea after prior ablation and subxiphoid drainage. Echocardiogram showed mildly dilated left atrium and preserved LV function. ECG confirmed AF.
Epicardial ablation	Robotic-assisted left chest epicardial ablation was performed. Adhesions were freed, and a lesion was created over the left PV. LOM was ablated, and LAA was clipped.
Discharge	Uneventful recovery and discharged.
Endocardial ablation	Endocardial catheter ablation was performed, which included PV isolation, extending the roof line, and additional substrate modifications.
Discharge	Uneventful recovery and discharged.
3 mo later	Clinical well and sinus rhythm on Holter monitoring.

AF = atrial fibrillation; ECG = electrocardiogram; LAA = left atrial appendage; LOM = ligament of Marshall; LV = left ventricular; PV = pulmonary veins.
